# Immunogenicity of targeted lentivectors

**DOI:** 10.18632/oncotarget.1680

**Published:** 2014-01-19

**Authors:** Cleo Goyvaerts, De Groeve Kurt, Sandra Van Lint, Carlo Heirman, Jo A. Van Ginderachter, Patrick De Baetselier, Geert Raes, Kris Thielemans, Karine Breckpot

**Affiliations:** ^1^ Laboratory of Molecular and Cellular Therapy, Department of Immunology-Physiology, Vrije Universiteit Brussel, Brussels, Belgium; ^2^ Laboratory of Cellular and Molecular Immunology, Vrije Universiteit Brussel, Brussels, Belgium; ^3^ Laboratory of Myeloid Cell Immunology, VIB, Brussels, Belgium

**Keywords:** lentivector, targeting, antigen presenting cell, vaccine, antitumor immunotherapy

## Abstract

To increase the safety and possibly efficacy of HIV-1 derived lentivectors (LVs) as an anti-cancer vaccine, we recently developed the Nanobody (Nb) display technology to target LVs to antigen presenting cells (APCs). In this study, we extend these data with exclusive targeting of LVs to conventional dendritic cells (DCs), which are believed to be the main cross-presenting APCs for the induction of a TH1-conducted antitumor immune response. The immunogenicity of these DC-subtype targeted LVs was compared to that of broad tropism, general APC-targeted and non-infectious LVs. Intranodal immunization with ovalbumin encoding LVs induced proliferation of antigen specific CD4^+^ T cells, irrespective of the LVs' targeting ability. However, the cytokine secretion profile of the restimulated CD4^+^ T cells demonstrated that general APC targeting induced a similar TH1-profile as the broad tropism LVs while transduction of conventional DCs alone induced a similar and less potent TH1 profile as the non-infectious LVs. This observation contradicts the hypothesis that conventional DCs are the most important APCs and suggests that the activation of other APCs is also meaningful. Despite these differences, all targeted LVs were able to stimulate cytotoxic T lymphocytes, be it to a lesser extent than broad tropism LVs. Furthermore this induction was shown to be dependent on type I interferon for the targeted and non-infectious LVs, but not for broad tropism LVs. Finally we demonstrated that the APC-targeted LVs were as potent in therapy as broad tropism LVs and as such deliver on their promise as safer and efficacious LV-based vaccines.

## INTRODUCTION

Numerous strategies have been developed to stimulate the patients' immune system to reject cancer cells. Of these, immunization with *ex vivo* generated dendritic cells (DCs) loaded with tumor-associated antigens (TAAs) has shown promise [1–3]. In order to perform their stimulatory function, these DCs are further activated, for which a multitude of stimuli are available [4]. A major question that remains is whether these *ex vivo* generated DCs resemble a specific DC-subset that can be found *in vivo* and linked herewith whether these are the best equipped for cancer immunotherapy purposes [5, 6].

Several DC-subsets have been described in mice. Simplified, DCs are subdivided in conventional DCs (cDCs), plasmacytoid DCs (pDCs) and certain tissue-specific populations such as Langerhans' cells (LCs) in the epidermis. In addition, cDCs are usually subdivided into CD8alfa^−^ and CD8afla^+^ cDCs [6, 7]. These DC-subsets are endowed with distinct functions and it is believed that cDCs, in particular CD8alfa^+^ cDCs are key players in the activation of cancer-specific immunity. They produce large amounts of IL-12, as such enabling the polarization of naive CD4^+^ T cells towards a T helper 1 (T_H_1) phenotype [8]. This is critical as these T_H_1 cells have three main functions in the anti-cancer immune response: (1) DC licensing, (2) supporting CD8^+^ cytotoxic T lymphocyte (CTL) responses and (3) aiding directly in tumor rejection [9]. Moreover, CD8alfa^+^ cDCs are involved in cross-presentation, a critical process in spontaneous tumor cell rejection. Although subtle differences exist between the human and mouse immune system, it needs to be highlighted that the aforementioned DC-subsets are also found in humans. Here pDCs are characterized by the expression of blood-DC antigen (BDCA) 2 and 4, whereas cDCs either express BDCA1 (CD1c) or BDCA3 (CD141) [6]. Formerly, BDCA3^+^ cDCs were seen as the counterparts of the mouse CD8alfa^+^ cDCs as they efficiently cross-present TAAs to CTLs and as both depend on transcription factor BATF3 for their generation [10]. However, recently the BDCA1^+^ cDCs were shown to closer resemble mouse CD8alfa^+^ cDCs in terms of IL-12 secretion and cross-presentation [11]. Since it remains a major challenge to generate *ex vivo* high numbers of DCs that resemble a certain subset, an attractive alternative would be to target DC-subsets *in vivo*.

Various TAA-delivery systems have been developed, including autologous and allogeneic tumor cell lysates, proteins, peptides, DNA- and mRNA-based formulations. In general these are all safe but as no intrinsic immunogenic factors are present, the inclusion of adjuvants or immunomodulators are crucial for optimal DC activation as otherwise tolerance could be induced [12]. Recently, the development of particulate delivery systems was introduced, in which TAAs are targeted to DCs along with adjuvants. These delivery systems have been extensively reviewed elsewhere [13]. However, TAA-derived proteins and peptides have poor pharmacokinetic properties and are rapidly cleaved. Moreover, as these are exogenous antigens, only cross-presentation can lead to MHC I-mediated CTL induction. Therefore the use of TAA-encoding plasmid DNA or mRNA would be more interesting.

An attractive way to introduce nucleic acids in antigen presenting cells (APCs) is through viral transduction. In this regard, lentivectors (LVs) represent excellent tools for the delivery of viral RNA to APCs [14]. In addition, they ensure persistent transgene expression in the transduced APC, which leads to continual antigen presentation and immunization [15]. Moreover, LVs are intrinsically immunogenic, which leads to activation of innate viral sensing pathways such as Toll like receptors (TLRs), which consequently leads to the induction of strong adaptive immunity [16–18]. In comparative studies of *in vivo* administration of LVs to *ex vivo* transduced DCs, peptide or DNA vaccination strategies, stronger TAA-specific immune responses were elicited with increased protection to tumor challenge and survival when immunization was performed with LVs [19–21]. This could be partially explained by the observation that cytokine driven DCs are less potent than DCs activated through microbial/viral signals in the generation of adaptive immunity [22]. Another important advantage of LVs is the ease with which their envelope can be engineered to alter their tropism. This process is called pseudotyping and enables targeting of specific DC-subsets. The latter is advantageous as it reduces the risk of insertional mutagenesis, since proviral DNA is only inserted in the genome of terminally differentiated APCs, which are short-lived after activation.

To answer the purpose of DC-subset specific targeting, we developed the Nanobody (Nb) display technology in which DC-specific Nbs are used as targeting moieties. These are inserted in the LV envelope together with a truncated version of the vesicular stomatitis virus glycoprotein (VSV.GS). This envelope initiates fusion after the Nb binds its antigen on the target cell. Previously we published the proof-of-principle of this technology in which we demonstrated that it is possible to produce Nb displaying LVs at high titers and that these allow *in vivo* targeting of APCs [23]. We used Nb DC2.1, which was described by De Groeve *et al.* to recognize a wide range of myeloid cells [24]. In the study, they also reported on Nb DC1.8, which specifically binds to immature bone marrow–derived DCs *in vitro*. In the present study we compared the *in vivo* transduction profile and immune stimulatory potential of broad tropism LVs to non-infectious BCII10, APC-targeted DC1.8 and DC2.1 displaying LVs, referred to as VSV.G-, BCII10-, DC1.8- and DC2.1-LVs respectively. We report on the possibility to exclusively transduce cDCs by DC1.8-LVs while also macrophages and pDCs are transduced by DC2.1-LVs. This difference in transduction profile was reflected in their potential to stimulate both antigen-specific CD8^+^ and CD4^+^ T cells. Surprisingly, the APC-targeted LVs as well as the non-infectious BCII10-LVs showed a similar therapeutic potential to VSV.G-LVs but for the former LV-types, this depended entirely on type I interferon (IFN).

## RESULTS

### DC1.8- and DC2.1-LVs target distinct mouse APC-subsets

Recently, we delivered a proof-of-principle on the Nb display technology, demonstrating high titer production of DC2.1-LVs and their ability to target mouse DCs and macrophages *in vivo* [23]. In this study we additionally utilized Nb DC1.8, which was shown to exclusively bind immature mouse DCs [24], demonstrating that also DC1.8-LVs can be produced at high titers (data not shown).

**Figure 1:**
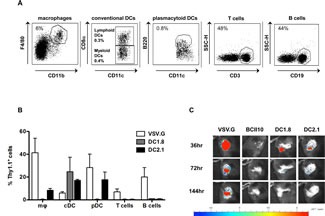
Targeted transduction of murine APCs *in situ* (A) To evaluate Thy1.1 expression in macrophages, cDCs, pDCs, B and T cells, LN cells were stained with an anti-Thy1.1 antibody in combination with antibodies directed against CD11b, F4/80, CD11c, B220, CD19 and CD3, respectively. (B) To track the transduced LN cells, we administered 15 μl of PBS (mock) or 10^E^6 TUs of the different LV pseudotypes encoding Thy1.1 to the inguinal LN of Thy1.2^+^ C57BL/6 mice. After 36 hours the injected LNs were isolated and flow cytometry was performed on single cell suspensions. The graph summarizes the results of 3 independent experiments as mean ± SEM (n = 3, 1 mouse per experiment). (C) To evaluate the transduction profile and kinetics of VSV.G-, BCII10-, DC1.8- and DC2.1-LVs *in vivo*, we injected 10^E^6 TUs of each LV stock encoding Fluc in the inguinal LN of C57Bl/6 mice. Thirty-six, 72 and 144 hours later, *in vivo* bioluminescence imaging was performed to obtain bioluminescent pseudo-color images superimposed on gray-scale photographs in which high luminescence is shown in red and weak luminescence in blue (n = 3, 1 mouse per experiment). The color scale underneath the images represents the correlation between the luminescent signal and the absolute amount of counts (light units).

To compare the transduction profile of DC1.8-LVs to that of VSV.G-, BCII10- and DC2.1-LVs *in vivo*, Thy1.1 encoding LVs were administered intranodally in C57BL/6 mice. Thirty-six hours later, LNs were resected and Thy1.1 expressing cells characterized by flow cytometry (Figure 1A), demonstrating that VSV.G-LVs transduced B and T cells, macrophages, cDCs and pDCs. In contrast, BCII10-LVs did not infect these cell types. Importantly, DC1.8-LVs specifically transduced CD8alfa^+^ and CD8alfa^−^ cDCs, while DC2.1-LVs additionally transduced macrophages and pDCs (Figure 1B). *In vivo* bioluminescence imaging was performed at several time points after intranodal delivery of FLuc encoding LVs to evaluate the persistence of modified cells. Thirty-six hours after administration of infectious LVs, expression of FLuc was observed as a luminescent signal, while no signal was observed after BCII10-LV injection. Luminescence was detectable for over 20 days when VSV.G-LVs were administered. In contrast, FLuc expression decreased to undetectable levels over a period of four days when DC1.8- or DC2.1-LVs were used (Figure 1C).

### Immunization with targeted LVs elicits CD8^+^ T lymphocyte responses, be it to a lesser extent than immunization with broad tropism LVs

**Figure 2:**
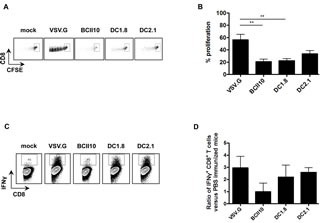
Intranodal delivery of targeted LVs encoding ovalbumin results in stimulation of ovalbumin-specific CD8^+^ T cells (A and B) CFSE-labeled OT-I cells were adoptively transferred to C57BL/6 mice one day prior to intranodal administration of PBS, 5x10^E^5 TUs VSV.G-, BCII10-, DC1.8- or DC2.1-LVs encoding ovalbumin. Five days later, LNs were resected, single cell suspensions prepared, after which induction of OT-I proliferation was evaluated by flow cytometry. Average values of the PBS group were substracted from the other values (n = 3, 2 mice per experiment). (C and D) To recover a reliable amount of CD45.2^+^ OT-I cells we adoptively transferred them to CD45.1^+^ C57BL/6 mice in which the CD45 marker served as a tool to distinguish which CD8^+^ T cells were OT-I and which were endogenous cells. Five days after immunization, the CD45.2^+^ fraction was isolated from single cell suspensions, which were subsequently restimulated with peptide loaded CD45.1^+^ splenocytes. After 72 hours, the amount of IFN-gamma ^+^ CD8^+^ T cells was evaluated in flow cytometry. Average values of the PBS group were substracted from the other values (n = 2, 2 mice per experiment).

Since CD8^+^ CTLs play a critical role in the control of cancer, we first evaluated the proliferation of CD8^+^ T cells upon immunization with ovalbumin encoding LVs. We transferred CD45.2^+^ OT-I cells to CD45.1^+^ mice after which these were immunized intranodally. Five days later, the injected LNs were isolated to evaluate OT-I proliferation in flow cytometry. Although proliferation of CD8^+^ T cells was observed in mice immunized with BCII10-, DC1.8- or DC2.1-LVs, it did not reach the levels observed in mice immunized with VSV.G-LVs (Figure 2A-B). To further investigate the ability of the targeted LVs to elicit functional CD8^+^ T cell responses, the percentage of IFN-gamma^+^ CD8^+^ T cells was evaluated by enriching the CD45.2^+^ OT-I cells from the injected LN and spleen, and *in vitro* restimulating them for 72 hours. This experiment demonstrated comparable percentages for VSV.G-, DC1.8- and DC2.1-LVs while this percentage decreased by half for BCII10-LVs (Figure C-D).

### The generation of antigen-specific CTLs upon immunization with targeted LVs depends on type I IFNs

**Figure 3:**
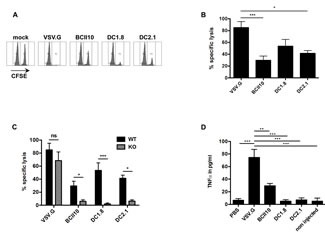
Intranodal delivery of targeted LVs encoding ovalbumin results in stimulation of cytotoxic ovalbumin specific CD8^+^ T cells and depends entirely on type I IFN Ovalbumin-specific cytotoxicity of the induced CD8^+^ T cells was evaluated *via* an *in vivo* CTL assay. This assay was performed in wild type (A and B) or in IFN type I receptor (IFNAR) ko mice (C), five days after intranodal administration of PBS, 5x10^E^5 TUs VSV.G-, BCII10-, DC1.8- or DC2.1-LVs encoding ovalbumin. Average values of the PBS group were substracted from the other values (n = 2, 3 mice per experiment). To evaluate the induction of the innate cytokine TNF-alfa, LNs were isolated and placed in culture three hours after they were injected with nothing, PBS, broad tropism, BCII10- or targeted-LVs. Twenty hours later, the supernatants of the crushed LNs were screened for the presence of TNF-alfa *via* ELISA (D) (n=2, 2 mice per experiment).

To evaluate the cytotoxic potential of antigen-specific CD8^+^ T cells, we performed an *in vivo* cytotoxicity assay. Although lysis of target cells was observed in mice immunized with DC1.8- or DC2.1-LVs, it did not reach the levels observed in mice immunized with VSV.G-LVs. In addition, as observed in the OT-I proliferation assay, immunization with BCII10-LVs also resulted in a certain degree of CTL induction (Figure 3A-B). Since pDCs and cDCs produce type I IFNs upon infection with VSV.G-LVs [11, 17], we addressed their role in the induction of ovalbumin-specific CTLs. Therefore we performed a CTL assay in IFNAR ko mice. The CTL response after administration of VSV.G-LVs was not significantly affected, whereas the CTL response after administration of DC1.8- and DC2.1-LVs as well as BCII10-LVs was dependent on type I IFNs (Figure 3C). We hypothesized that this might be explained by a different activation of the innate immune system by the broad tropism LVs. Therefore we evaluated the induction of TNF-alfa as this cytokine was previously described to be upregulated upon systemic delivery of broad tropism LVs. We observed that the production of TNF-alfa was most pronounced upon delivery of broad tropism LVs, which strengthens the hypothesis that broad tropism and targeted LVs use other innate cytokines such as TNF-alfa and type I IFN, respectively (Figure 3D).

### Immunization with targeted LVs elicits distinct antigen-specific CD4^+^ T cell responses

**Figure 4:**
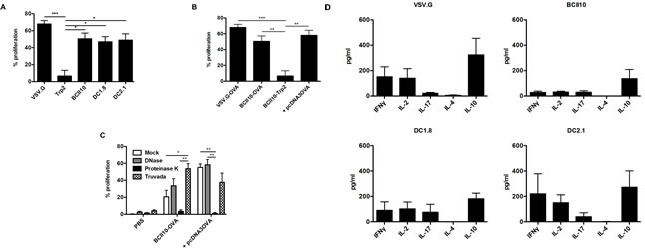
Intranodal delivery of targeted as well as BCII10-LVs encoding ovalbumin results in stimulation of ovalbumin-specific CD4^+^ T cells (A-C) CFSE labeled OT-II cells were adoptively transferred one day prior to intranodal administration of PBS or 5x10^E^5 TUs of the respective LVs. Five days later, LNs were resected and single cell suspensions prepared to evaluate the induction of OT-II proliferation by flow cytometry. Average values of the PBS group were substracted from the other values (n = 2, 2 mice per experiment). (D) Alternatively, CD4^+^ T cells were sorted out of the LN-derived single cell suspensions and restimulated with ISQAVHAAHAEINEAGR peptide-loaded bone marrow-derived DCs for 48 hours after which supernatants were analyzed for their presence of IFN-gamma, IL-2, IL-17, IL-4 and IL-10 in ELISA. Average values of the PBS group were substracted from the other values (n = 3, 4 mice pooled in two groups per experiment). More specifically in (A) VSV.G-, BCII10-, DC1.8- or DC2.1-LVs encoding ovalbumin as well as VSV.G-LVs encoding Trp2 were injected, in (B) VSV.G- and BCII10-LVs encoding ovalbumin, BCII10-LVs encoding Trp2 or BCII10-LVs encoding Trp2 produced in the presence of an ovalbumin encoding plasmid (depicted as + pcDNA3OVA) were administered and in (E) BCII10-LVs encoding ovalbumin or Trp2 + pcDNA3OVA and pretreated with 10 U/ml DNase, 100 μg/ml Proteinase K were used or mice were injected intraperitoneally 24 hours earlier with 5 ng of the RT inhibitor, Truvada.

Although CTLs are considered to be the main effector cells in anti-tumor immunity, accumulating evidence shows that CD4^+^ T cells are another critical component [25, 26]. Therefore, we evaluated the expansion of ovalbumin-specific CD4^+^ T cells upon intranodal immunization. Proliferation of OT-II cells was strongest after immunization with VSV.G-LVs. Notably, the proliferation of OT-II cells was comparable after administration of targeted as well as BCII10-LVs (Figure 4A). To ensure the specificity of this response, we immunized mice with VSV.G-LVs encoding Trp2, demonstrating a lack of proliferation. As BCII10-LVs are unable to transduce APCs, we hypothesized that these viral particles were engulfed *via* an alternative route, resulting in the uptake of the ovalbumin encoding viral genome or possibly plasmid DNA or proteins [27]. To evaluate this hypothesis, we produced BCII10-LVs encoding Trp2 in the presence of an ovalbumin encoding eukaryotic expression vector. This allows non-specific packaging of plasmid DNA and proteins [28]. Subsequently, mice were immunized with these LVs. Strong proliferation of OT-II cells was induced (Figure 4B). Next we scrutinized whether this proliferation was due to the presence of plasmid DNA or protein. Therefore, mice were immunized with LVs that were pre-treated with DNase or proteinase K to remove plasmid DNA or proteins respectively. To investigate the role of the viral genome, mice were injected 24 hours prior to immunization with the RT inhibitor Truvada [29]. Proliferation of ovalbumin-specific CD4^+^ OT-II cells was observed in all conditions, except when LVs were pre-treated with proteinase K suggesting that the presence of protein within the LV stock is the main inducer of OT-II proliferation (Figure 4C).

Since administration of BCII10-LVs was sufficient to induce antigen-specific CD4^+^ T cell proliferation, but had only minor effects on CD8^+^ T cell induction when compared to targeted LVs, we addressed which CD4^+^ T cell phenotype was stimulated by the LVs. Therefore we evaluated the secretion of cytokines by CD4^+^ T cells that were restimulated *in vitro*. The CD4^+^ T cells from mice immunized with VSV.G- and DC2.1-LVs induced a similar T_H_1 profile with similar amounts of IFN-gamma, IL-2, and IL-10. To our surprise, mice immunized with DC1.8-LVs produced less of these cytokines, which made their profile similar to the one induced by the BCII10-LVs (Figure 4D).

### Immunization with broad tropism or targeted LVs results in similar therapeutic efficacy

**Figure 5:**
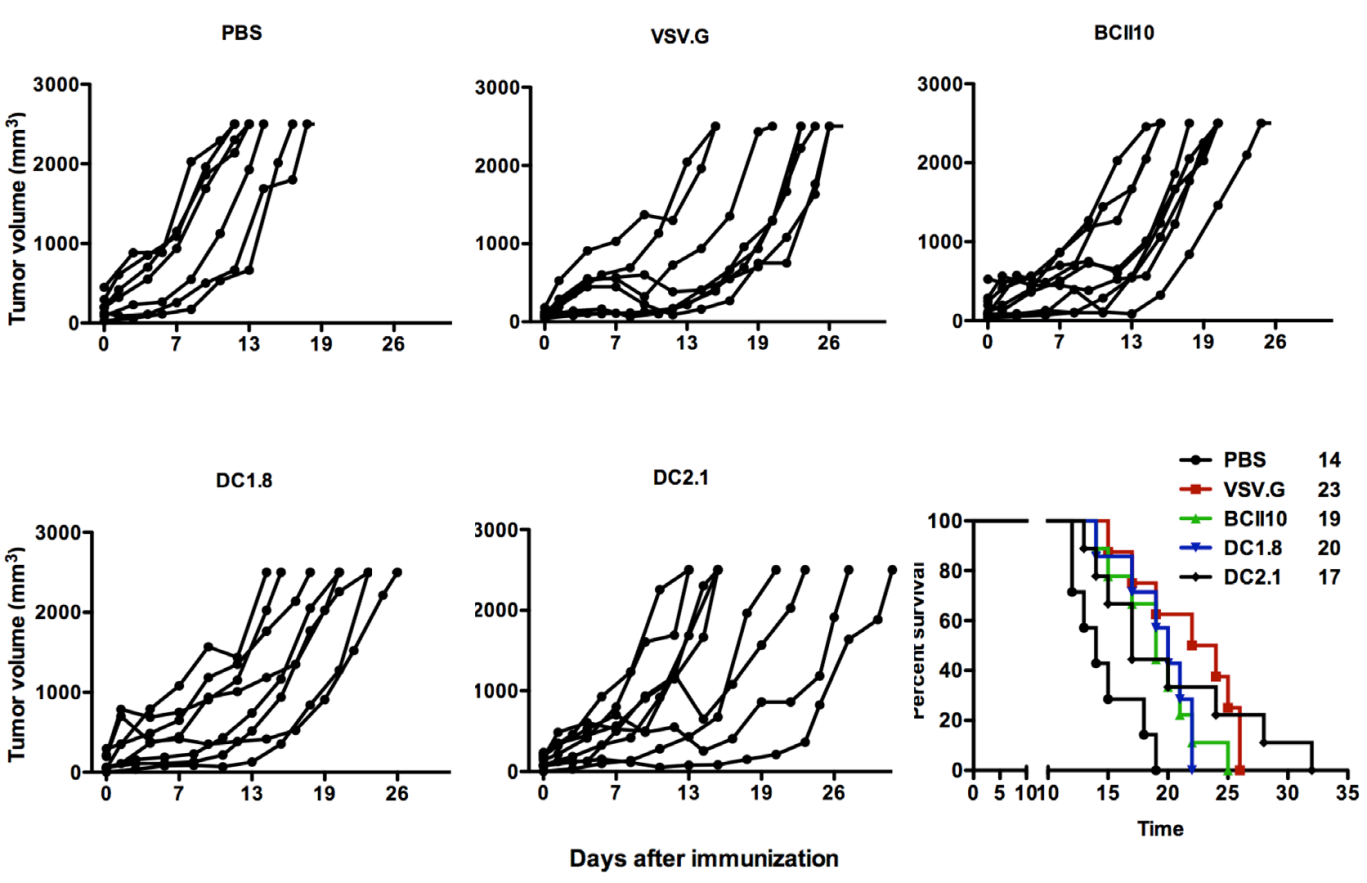
Intranodal vaccination of targeted LVs encoding ovalbumin results in prolonged survival in an E.G7-OVA model To evaluate the therapeutic potential of the targeted LVs, C57BL/6 mice were challenged on day 0 with 3x10E5 E.G7-OVA cells. When palpable tumors were present mice were immunized intranodally with PBS or 10E6 TUs VSV.G-, BCII10-, DC1.8- or DC2.1-LVs encoding ovalbumin. Seven days later, the treatment was repeated. Tumor growth and survival were examined every two days. The results shown are representative for two independent experiments (n = 2, 4 mice per experiment).

To evaluate the therapeutic potential of the LVs, mice bearing E.G7-OVA tumors were immunized twice at a seven-day interval. The median survival of mice treated with PBS was 14 days while this was prolonged to 19, 20, 17 and 23 days upon immunization with ovalbumin encoding BCII10-, DC1.8-, DC2.1- and VSV.G-LVs respectively. Of note, although DC2.1-LVs induced the lowest median survival in days, they did show the longest dispersion in time with the last mouse surviving till day 32 (Figure 5).

## DISCUSSION

Targeting APCs is believed to be key to develop safe and efficacious LV-based anti-cancer vaccines [30–34]. Here we report on the Nb display technology to target LVs to mouse APC-subsets.

We compared the transduction profile of broad tropism LVs to LVs harboring the β-lactamase-specific Nb BCII10, or the APC-targeting Nbs DC1.8 or DC2.1. We chose to deliver these LVs to the LN as this organ contains a relative high number of DCs [35] and as it has been described that lower antigen doses are required to achieve similar immune responses [36]. Tracking the transduced cells within the LN demonstrated the exclusive transduction of cDCs by DC1.8-LVs, while DC2.1-LVs transduced cDCs, pDCs and macrophages. Of note, both the CD8alfa^+^ and CD8alfa^−^ cDC fractions were transduced by these LVs. The VSV.G-LVs additionally transduced B and T cells which is expected as VSV.G is the envelope of choice for efficient transduction of a broad range of cells [37]. Of note, it has been postulated that targeting LVs would increase their transduction efficiency [38]. However, similar to the observations of Ageichik *et al*., who targeted LVs to MHC II-expressing cells [33], we observed a lower transduction efficiency with DC1.8- and DC2.1-LVs compared to VSV.G-LVs. In contrast, Yang *et al.* observed that LVs targeted to DC-SIGN were more efficient in transduction of CD11c^+^ cells than VSV.G-LVs [39]. The latter might be explained by the type of antigen targeted, both in terms of its expression level and function. For instance, DC-SIGN is a C-type lectin, involved in the capture of HIV-1 and furthermore protects it from degradation in early endosomes [40]. The results from the *in vivo* bioluminescence imaging upon delivery of FLuc encoding LVs, support the notion that only short-lived APCs are transduced by DC1.8- and DC2.1-LVs. As expected, the luminescence signal lasted for more than 20 days after VSV.G-LV delivery, implying the transduction of long-lived, possibly non-APCs, which increases the risk of insertional mutagenesis [41].

Because DC1.8- and DC2.1-LVs deliver their cargo to different APC-subsets, we evaluated and compared their immunogenicity. It is generally accepted that cDCs within the LNs are required to present antigens to CD8^+^ T cells, while migrated DCs and pDCs are insufficient to prime T cells [42, 43]. Moreover within the cDC population it was found that the CD8alfa^+^ DEC205^+^ DC population surpasses other APC-subsets in cross-presenting skills [44–47]. Fuertes *et al.* further demonstrated that host type I IFNs are critical for the innate immune recognition of a growing tumor through signalling on the CD8alfa^+^ DC fraction [48]. In line with this observation, it was described that cDCs in a human setting need type I IFN and TNF-alfa, released from non-replicating HIV-1 activated pDCs, to induce their full bystander maturation [49, 50]. Although cDCs seem to have a prominent role, the latter study stresses the importance of the pDCs. However, it was also shown in certain tumor models that tumor draining LN-resident pDCs expressed the enzyme indoleamine 2,3-dioxygenase, which induces the generation of regulatory T cells [51, 52]. The role of LN-resident macrophages in T cell stimulation is less defined. Although it was shown that CD8^+^ T cells interact with virally infected macrophages and DCs, macrophages were not shown to fully prime anti-viral CD8^+^ T cells [35]. Moreover, Albert *et al.* showed that human DCs but not macrophages efficiently presented antigens from apoptotic cells to CD8^+^ T cells [53]. Nonetheless, some studies report that macrophages can cross-prime CD8^+^ T cells [54]. In order to shed light on the importance of macrophages and pDCs in the stimulation of T cell responses, we evaluated the expansion of CD4^+^ and CD8^+^ T cells as well as their functionality upon immunization with DC1.8-, DC2.1- and VSV.G-LVs. Although immunization with VSV.G-LVs elicited stronger CD8^+^ T cell responses, we demonstrated that DC1.8- and DC2.1-LVs were equally potent in the induction of CD8^+^ T cells with lytic and more importantly therapeutic efficacy. Interestingly, we observed that the induced CD4^+^ cytokine profile of DC2.1-LVs closely resembled that of VSV.G-LVs as both elicited a similar amount of IFN-gamma, IL-2 and IL-10. These cytokines are typical for a T_H_1 response and therefore crucial for a potent antitumor immune response. In contrast, DC1.8-LVs induced less of these cytokines while its profile resembled more that of the non-infectious BCII10 LVs, suggesting that presentation of antigens by cDCs alone is probably not as adequate as presentation by a broad range of APCs. Another striking observation was the complete abrogation of CTL inducing capacity of DC1.8- and DC2.1-LVs in IFNAR ko mice. The latter was unexpected, as it was described that pDCs and LN-resident macrophages are the main producers of type I IFNs upon LV transduction [35, 55]. Therefore, we only expected to observe a reduction in immunogenicity in IFNAR ko mice for VSV.G- and DC2.1-LVs. However, the immunogenicity of VSV.G-LVs was unaffected, whereas that of DC2.1- and DC1.8-LVs was compromised. The latter can in part be explained by our previous observations that LVs activate TLR3/7 and as such induce type I IFN production by DCs [17]. The ability of VSV.G-LVs to elicit CTL responses in IFNAR ko mice suggests that these activate other innate pathways that are able to bypass the need for type I IFNs. The latter hypothesis was strengthened by the observation that VSV.G-LVs triggered the highest production of the pro-inflammatory cytokine TNF-alfa [49, 56].

Another surprise was the ability of the BCII10-LVs to induce immune responses, despite their inability to transduce cells. The latter was explained by the presence of protein contaminants within the LV preparation and highlights the need for the development of easy to use and high efficiency LV-purification systems. Although, immunization with BCII10-LVs resulted in T cell responses, it is important to note that the CD4^+^ and CD8^+^ T cell responses upon immunization with infectious LVs were stronger. These observations are in line with the observations of Ageichik *et al*., who compared the induction of IFN-gamma^+^ CD8^+^ T cells upon administration of LVs targeted to MHC II or carcinoembryonic antigen [33]. However, in therapy, no striking differences were observed between BCII10-, DC1.8-, DC2.1- and VSV.G-LVs. The latter has not been described but this could be explained by the fact that previous reports on the use of targeted LVs for anti-cancer vaccination only compared LVs encoding the tumor antigen versus LVs encoding an irrelevant antigen [39, 57]. Importantly, studies using nanoparticles demonstrate that the strength of the immune responses induced is co-determined by the cell population that engulfs the particle as well as the cargo of these particles [58, 59]. We delivered the BCII10-LVs intranodally, which can be considered as an anatomical targeting of virus like particles [31]. It is therefore likely that BCII10-LVs have been engulfed by DCs and as such deliver proteins as well as viral components, *ie* activation signals which ultimately led to the induction of a small amount of TNF-alfa, IFN type I and IL-10.

In conclusion, we demonstrate that LV-targeting to distinct DC-subsets *via* the Nb display technology is feasible and induces qualitatively distinct T cell responses that are equally potent in the control of cancer. Furthermore, this targeting strategy increases the LV-based vaccines' safety through faster LV-clearance. In the future, experiments with tumor relevant antigens like MageA3, Trp2, p62 and brachyury, have to confirm the observed results with the egg white derived protein ovalbumin [60–63]. Furthermore the possible relief of side effects like vitiligo [64] with APC-targeted LVs has to be evaluated. Since we possess Nbs that target human cDCs alone [65] or human cDCs, pDCs and macrophages [23], this method represents a good option to produce LV-based vaccines for preclinical studies in cancer.

## MATERIALS AND METHODS

### Mice and cell cultures

Six to 12 week old female CD45.2^+/^Thy1.2^+^ C57BL/6 mice were purchased from Harlan. The CD45.1^+^/Thy1.2^+^ C57BL/6 mice were obtained from J. Van Ginderachter (Vrije Universiteit Brussel). Transgenic OT-I and OT-II mice were purchased from Charles River. Mice lacking the IFN type I receptor (IFNAR ko) were obtained from C. Libert (Universiteit Gent). All animals were handled according to the institutional guidelines and experiments were approved by the Ethical Committee for use of laboratory animals of the Vrije Universiteit Brussel. The human embryonal kidney 293T cell line (HEK293T) and T-cell lymphoma cell line E.G7-OVA were cultured as recommended by the American Type Culture Collection (ATCC). Single cell suspensions were prepared from spleens and lymph nodes (LNs) as described [23].

### Lentiviral vector production

Plasmids. The packaging plasmid pCMVΔR8.9 and VSV.G encoding plasmid pMD.G were a gift from Dr. D. Trono (University of Geneva). The plasmid pUB6-VSV.GS, which encodes the binding-defective, but fusion-competent VSV.G was described by Zhang *et al.* [66]. The plasmids pSIN-Thy1.1, pHR trip CMV luc2-Ires-tNGFR SIN, pHR' trip CMV Ii80tOVA-Ires-tNGFR SIN, pcDNA1 Ii80tOVA, pHR' trip CMV Nb BCII10 SIN and pHR' trip CMV Nb DC2.1 SIN were described [17, 23, 28, 67]. The sequence encoding the membrane bound form of Nb DC1.8 was cloned into the pHR' trip CMV SIN vector following the same strategy as described for Nb BCII10 and DC2.1 [23].

Virus production and characterization. Production of broad tropism and targeted LVs as well as their characterization was performed as described [23].

### *In vivo* bioluminescence imaging

*In vivo* bioluminescence imaging was performed at the indicated time points to visualize *in situ* transduction of cells after intranodal delivery of 10^E^6 transducing units (TUs) of FLuc encoding LVs. The procedure was performed as described [68].

### *In vivo* tracking of lentivirally transduced cells

To evaluate the transduction profile of the LVs, C57BL/6 mice were injected in the inguinal LN with 10^E^6 TUs of Thy1.1 encoding LVs. Thirty-six hours later, mice were euthanized, injected LNs isolated, single cell suspensions prepared and cells characterized by flow cytometry [23].

### *In vivo* OT-I and OT-II stimulation with ELISA on restimulated OT-II cells

One day prior to immunization, 1-2 x 10^E^6 purified and carboxyfluorescein diacetate succinimidine ester (CFSE, Invitrogen) labeled CD8^+^ OT-I or CD4^+^ OT-II spleen cells were adoptively transferred to mice by intravenous injection. Mice were immunized through single administration of 5 x 10^E^5 TUs of broad tropism or Nb-targeted LVs encoding ovalbumin into the inguinal LN. Administration of PBS or BCII10-LVs served as a control. Five days post-immunization, proliferation of T cells was analyzed. Therefore, LNs were isolated, single cell suspensions prepared, stained with a peridinin-chlorophyll protein-Cy5.5 (PerCP-Cy5.5) conjugated antibody against CD8 or CD4 (BD) and analyzed *via* flow cytometry as described [20]. When mice were adoptively transferred with OT-II cells, part of the single cell suspensions were used to sort and restimulate the CD4^+^ T cells. Therefore, 5 x 10^E^5 of CD4^+^ T cells were plated in a 48-well plate in 500 μl of culture medium and restimulated for 48 hours with bone marrow-derived DCs that were loaded for two hours with 5 μM ISQAVHAAHAEINEAGR peptide (Eurogentec) at a 10:1 ratio. Supernatants were screened in enzyme-linked immunosorbent assay (ELISA) for the presence of IFN-gamma, IL-2, IL-17, IL-4 and IL-10 (eBioscience).

### Intracytoplasmic staining of IFN-gamma

One day prior to immunization, CD45.1^+^ C57BL/6 mice were injected with 5 x 10^E^6 CD45.2^+^ OT-I cells. Mice were immunized as described above. Five days post-immunization, spleens and LNs were isolated and CD45.2^+^ OT-I cells sorted using anti-CD45.2 beads (Miltenyi). Subsequently, 5 x 10^E^5 of these cells were plated in a 48-well plate in 500 μl of RPMI+ (RPMI-1640 medium supplemented with 5% FCI, 5% supplements and 50 μmol/L β-mercaptoethanol) and restimulated with SIINFEKL loaded CD45.1 splenocytes at a 10:1 ratio. Two days later Golgi plug was added (1mg/ml, BD) and intracytoplasmic expression of IFN-gamma was evaluated the next day by intracellular staining with a phycoerythrin-Cy7 (PE-Cy7) conjugated anti-IFN-gamma antibody (eBioscience) followed by flow cytometric analysis.

### *In vivo* cytotoxic T lymphocyte assay

Mice were immunized through intranodal administration of 5 x 10^E^5 TUs of broad tropism or targeted ovalbumin encoding LVs. Administration of PBS or BCII10-LVs served as a control. The *in vivo* cytotoxicity assay was performed five days post-immunization as described [20].

### Evaluation of TNF-alfa secretion after intranodal immunization

Mice were injected intranodally with 5 x 10^E^5 TUs of broad tropism or targeted ovalbumin encoding LVs. No injection, PBS or BCII10-LVs served as control. Three hours later, LNs were resected and transferred to a 24-well plate with 300 μl of RPMI+. Twenty hours later, supernatants were screened in ELISA for the presence of TNF-alfa (eBioscience).

### Flow cytometry

Staining of surface markers was performed as described [23]. Data were collected using a FACS Canto flow cytometer (BD) and analyzed using FACSDiva™ (BD) or FlowJo™ (Tristar Inc.) software.

### Tumor rejection experiments

Mice were grafted subcutaneously with 3 x 10^E^5 E.G7-OVA tumor cells at the tail base. Mice bearing palpable tumors were immunized twice with 10^E^6 TUs of LVs encoding ovalbumin at a seven-day interval. Tumor growth was monitored. Mice were killed when the tumor reached a volume of 2500 mm^3^.

### Statistical analyses

A one-way ANOVA followed by a Bonferroni's multiple comparison test was performed. Sample sizes and number of times experiments were repeated are indicated in the figure legends. Number of asterisks in the figures indicates the level of statistical significance as follows: *, *p* < 0.05; **, *p* < 0.01; ***, *p* < 0.001. The results are shown in a column graph or table as the mean ± SEM. Survival was visualized in a Kaplan-Meier plot. Differences in survival were analyzed by the log-rank test.
